# Virulence traits and novel drug delivery strategies for mucormycosis post-COVID-19: a comprehensive review

**DOI:** 10.3389/fimmu.2023.1264502

**Published:** 2023-09-25

**Authors:** Md. Faiyazuddin, A. Sophia, Sumel Ashique, Amol D. Gholap, S. Gowri, Sourav Mohanto, C. Karthikeyan, Sagnik Nag, Arif Hussain, Mohammad Shabib Akhtar, Md. Afroz Bakht, Mohammed Gulzar Ahmed, Sarvesh Rustagi, Alfonso J. Rodriguez-Morales, Luis Andres Salas-Matta, Aroop Mohanty, D. Katterine Bonilla-Aldana, Ranjit Sah

**Affiliations:** ^1^ School of Pharmacy, Al – Karim University, Katihar, Bihar, India; ^2^ Nano Drug Delivery®, Raleigh-Durham, NC, United States; ^3^ PG & Research Department of Physics, Cauvery College for Women (Autonomous), Tiruchirappalli, Tamil Nadu, India; ^4^ Department of Pharmaceutics, Pandaveswar School of Pharmacy, Pandaveswar, West Bengal, India; ^5^ Department of Pharmaceutics, St. John Institute of Pharmacy and Research, Palghar, Maharashtra, India; ^6^ Department of Pharmaceutics, Yenepoya Pharmacy College & Research Centre, Yenepoya (Deemed to be University), Mangalore, Karnataka, India; ^7^ Department of Chemical & Biochemical Engineering, Dongguk University, Seoul, Republic of Korea; ^8^ Department of Bio-Sciences, School of Biosciences & Technology (SBST), Vellore Institute of Technology (VIT), Tamil Nadu, India; ^9^ School of Life Sciences, Manipal Academy of Higher Education, Dubai, United Arab Emirates; ^10^ Department of Clinical Pharmacy, College of Pharmacy, Najran University, Najran, Saudi Arabia; ^11^ Chemistry Department, College of Science and Humanity Studies, Prince Sattam Bin Abdulaziz University, Al-Kharj, Saudi Arabia; ^12^ School of Applied and Life Sciences, Uttaranchal University, Dehradun, Uttarakhand, India; ^13^ Grupo de Investigación Biomedicina, Faculty of Medicine, Fundación Universitaria Autónoma de las Américas—Institución Universitaria Visión de las Américas, Pereira, Colombia; ^14^ Faculties of Health Sciences and Environmental Sciences, Universidad Científica del Sur, Lima, Peru; ^15^ Gilbert and Rose-Marie Chagoury School of Medicine, Lebanese American University, Beirut, Lebanon; ^16^ Department of Clinical Microbiology, All India Institute of Medical Sciences, Gorakhpur, India; ^17^ Research Unit, Universidad Continental, Huancayo, Peru; ^18^ Institute of Medicine, Tribhuvan University Teaching Hospital, Kathmandu, Nepal; ^19^ Department of Clinical Microbiology, DY Patil Medical College, Hospital and Research Centre, DY Patil Vidyapeeth, Pune, Maharashtra, India; ^20^ Datta Meghe Institute of Higher Education and Research, Jawaharlal Nehru Medical College, Wardha, India

**Keywords:** mucormycosis, COVID-19, infectious disease, virology, nanomedicine, diagnosis

## Abstract

The outbreak of a fatal black fungus infection after the resurgence of the cadaverous COVID-19 has exhorted scientists worldwide to develop a nutshell by repurposing or designing new formulations to address the crisis. Patients expressing COVID-19 are more susceptible to Mucormycosis (MCR) and thus fall easy prey to decease accounting for this global threat. Their mortality rates range around 32-70% depending on the organs affected and grow even higher despite the treatment. The many contemporary recommendations strongly advise using liposomal amphotericin B and surgery as first-line therapy whenever practicable. MCR is a dangerous infection that requires an antifungal drug administration on appropriate prescription, typically one of the following: Amphotericin B, Posaconazole, or Isavuconazole since the fungi that cause MCR are resistant to other medications like fluconazole, voriconazole, and echinocandins. Amphotericin B and Posaconazole are administered through veins (intravenously), and isavuconazole by mouth (orally). From last several years so many compounds are developed against invasive fungal disease but only few of them are able to induce effective treatment against the micorals. Adjuvant medicines, more particularly, are difficult to assess without prospective randomized controlled investigations, which are challenging to conduct given the lower incidence and higher mortality from Mucormycosis. The present analysis provides insight into pathogenesis, epidemiology, clinical manifestations, underlying fungal virulence, and growth mechanisms. In addition, current therapy for MCR in Post Covid-19 individuals includes conventional and novel nano-based advanced management systems for procuring against deadly fungal infection. The study urges involving nanomedicine to prevent fungal growth at the commencement of infection, delay the progression, and mitigate fatality risk.

## 1 Introduction

Mucormycosis (MCR) is one of the “opportunistic” fungi belonging to the *zygomycete* family (1, 2). The leading cause of infection is inhaling spores, which can spread to immune-compromised individuals’ paranasal sinuses and lungs, eventually resulting in rhino-cerebral disease - a primary type of mycosis in post-COVID-19 infection cases (3–5). MCR is non-pathological in the immune-competent population due to intact immunity via neutrophils. In contrast, it can cause severe invasive fungal infections in immune-compromised patients with uncontrolled diabetes mellitus (DM), diabetic ketoacidosis (DKA), wound injury (6), HIV/AIDS, cancer, and organ transplant (7, 8). Moreover, this “opportunistic” fungus can be clinically classified as cutaneous, disseminated, pulmonary, rhino-orbital, or gastrointestinal and is comprehended to cause renal infections, osteomyelitis, endocarditis, and peritonitis (9). Several published reports indicated that the risk factors for mycosis include immune suppression (10), DKA, DM, and corticosteroid administration (7–9, 11).

Nanotechnology provides an exceptional opportunity for medicines to improve their efficacy and delivery prospects (12). The pharmaceutical nanotechnology utilizes various delivery systems or carriers, i.e., nanoliposomes, nanocrystals, nanoemulsions, lipid nanocarriers, polymeric microspheres, nanosuspensions, dendrimers, poly(prodrug) amphiphile, cross-linked and smart hydrogel, monoclonal antibodies (mAbs)-tailored nanoparticles (NPs), site-specific nanocarriers, etc. to enhance the effectiveness of various conventional therapeutics or drug molecules in the modern era of diseases (13, 14). Nanotechnology implies the construction or encapsulation of low dosages of potent therapeutics in targeted organs and tissues with minimal or non-significant side effects (12). Various empirical investigations have shown that NPs penetrate the microbial cells, causing impairment or lysis. In addition, NPs can cause structural modifications in the cell membrane by forming pits on the microbial cell surface, accumulating nanoparticles in the microbial cells. These events resulted in changes in cell permeability and resulted in apoptosis. The free radicals also induce cell damage after the exposure of fungal cells to nanoparticles; protein and sugar content are significantly decreased, which disrupts the fungal hyphae (15–17). Various investigations revealed the probable mechanisms of metal oxide nanoparticles for antifungal activity. In general, nanoparticles release ions that bind to specific protein groups found in microbes, thus disrupting integral membrane proteins and interfering with cell permeability (18, 19). Additionally, NPs can prevent the germination of conidia and hinder their development. The existence of microbes interfered with the oxidative electron transport of proteins, while the nanoparticles increased gene transcription levels in response to oxidative stress, ultimately affecting the mitochondrial membrane. Different types of NPs can further trigger specific oxidation reactions, damaging proteins, DNA, and membranes and interfering with nutrient absorption via microbes (20–23).

**Figure 1:**
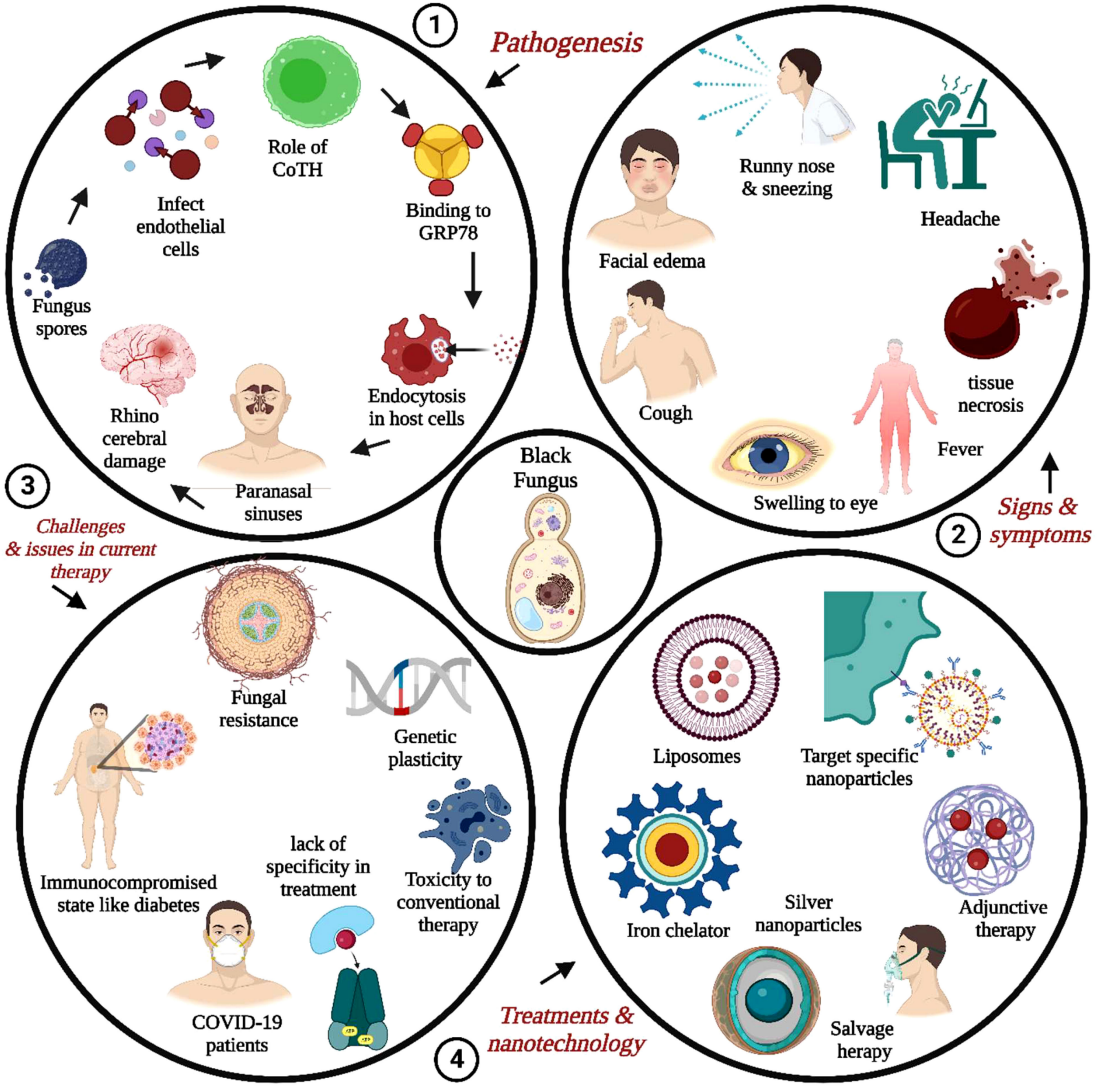
General overview or understanding of pathogenesis, virulence traits, signs and symptoms, and novel treatment approaches primarily related to nanomedicine or novel drug delivery systems for the infected patients of Mucormycosis (MCR), especially in post COVID-19 era.

Therefore, this review aims to disseminate information on pathogenesis, virulence traits, and novel treatment approaches for MCR in post-COVID-19. This co-occurring article also summarizes clinical manifestations and statistics of infected patients for MCR, especially in the COVID-19 era, and further recapitulates the overview of the article in **Figure 1**. It also covered the novel nanomedicines and the futuristic scopes for adequate managing invasive infections. The instantaneous medical attention is required if a patient suffers from painful lungs, sinuses, skin abrasion, or suspected infection. During diagnosis, the healthcare practitioners must consider the patient’s medical history, current bothersome symptoms, and laboratory testing.

## 2 Phyla of pathogenic causative fungi

The MCR in post-COVID-19 is a rare infection and highly prevalent in natural disaster-prone areas (24, 25). Worldwide, 1.7 per 100,000 people have been infected by this disease (26), while in India, it has been about 80 times more rampant than in Western countries (24). Several countries have reported MCR as a comorbidity associated with COVID-19. The condition is often called “*black fungus*” since fungi-infested tissue appears black (27). Various fungi DNA have been encountered in numerous substrates, i.e., spacecraft, soil, wood, water, manure, decaying fruits, and vegetables (24, 26). Mucorales fungi (7, 28) are distinguished by filamentous hyphae that produce airborne fungal spores known as conidia, further aid in the propagation of the fungal species (29, 30). Zygomycota, the causative agent of MCR, is now classified in a new phylum, Glomeromycota, the order Mucorales falls under the subdivision mucoromycotina (1). After Aspergillus, this fungus has emerged as one of the most problematic pathogens in recent years, causing invasive diseases in patients with new coronavirus infections and diabetes (31–33). The infection known as MCR is a result of fungi belonging to the Mucorales order. Apophysomyces, a different family member that thrives in tropical and subtropical climes, is also widespread in India. About 70% of the cases are driven by *Rhizopus oryzae*, a significant cause of MCR, a severe and often life-threatening fungal infection. However, it is necessary to elucidate that while Rhizopus oryzae is a commonly associated organism with MCR, it might not be accurate to label it as the “most fundamental organism causing fungal infections.” The prevalence of different causative agents for MCR might vary based on geographic location, patient population, and other factors. While *Rhizopus oryzae* might be responsible for a significant portion of MCR cases, other fungi within the Zygomycetes class can also cause these infections. Many different types of fungal infections are caused by a wide range of fungal species, each with varying levels of severity and clinical significance (34, 35). The different species of fungal phyla, pathogenic to humans, causing MCR are tabulated in **Table 1**. The review suggests that the novel formulations discussed against MCR can be tested against these fungal pathogens listed in the **Table 1**.

**Table 1:** Description of various pathogens that causes mycosis.

Phylum: Glomeromycota	
Subphylum: Mucoromycotina *(Incertae sedis)* Order: Mucorales (mucormycosis)	*Absidia corymbifera* *Apophysomyces elegans* *Apophysomyces variabilis* *Apophysomyces trapeziformis* *Mucor indicus* *Rhizomucor pusillus* *Rhizopus oryzae* *Cunninghamella bertholletiae* *Cokeromyces recurvatus* *Saksenaea vasiformis* *Syncephalastrum racemosus*

## 3 Pathogenesis of mucormycosis

In early 1876, a cancer patient in Germany was diagnosed with hemorrhagic pulmonary infarction, confirmed to be caused by sporangia and fungal hyphae, marking the first recorded case of MCR (36). The World Health Organization (WHO) has reported that MCR is significantly more prevalent in India than in other parts of the world, with estimated rates ranging from 0.02 to 9.5 cases per 100,000 individuals. However, due to a lack of population-based data, it is challenging to determine the accurate incidence and prevalence of MCR in India (37). In a recent year, a computational model suggests that the number of cases of MCR in India is around 14 per 100,000 people (26, 38). The general population is vulnerable to evading fungal spores against host-defence mechanisms due to immunodeficiency and macrophage deductions (39), getting attached to host tissues and growing saprophytically. After infection, the fungus exhibits fungal dimorphism and proliferates uncontrollably under favorable conditions, making it difficult to control its growth and progression. Monoclonal antibodies are utilized to treat COVID-19; meanwhile, other therapies like tocilizumab, casirivimab, imdevimab, and regdanvimab lead to immune suppression, succumbing to the infected patients (40). The infection may further target the body’s major organs, including the brain, lungs, and kidneys (41). Several clinical investigations further confirmed the involvement of eyes, skin, and sinuses, which are severely affected by this fatal infection, leading to significant symptoms or signs of swollen eyes, runny nose, hazy vision, and facial swelling (41).

**Figure 2:**
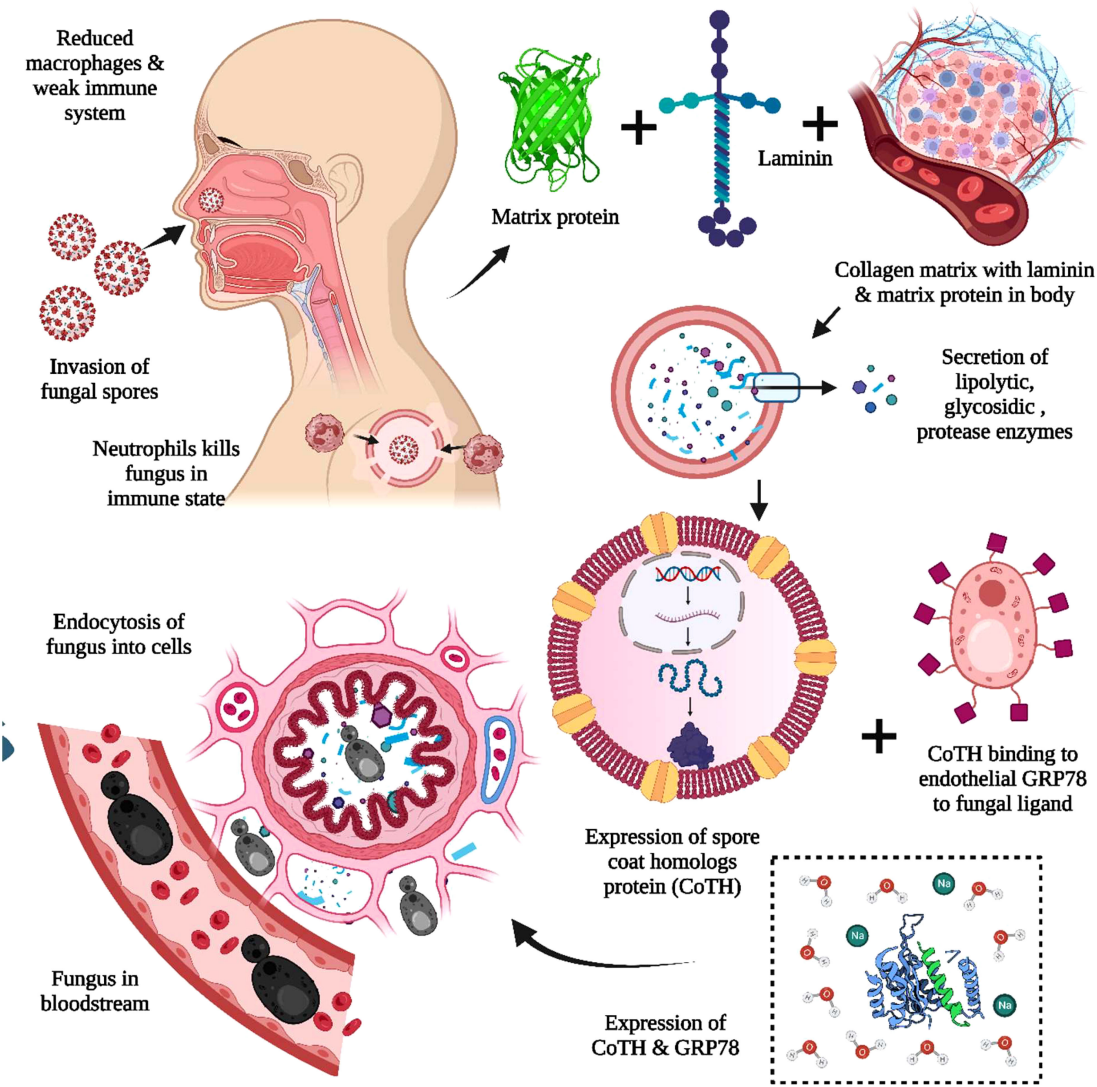
The pathogenesis of Mucormycosis (MCR). The reduced macrophages and weak immune systems play crucial roles in invading black fungus. The interaction with the host cell is facilitated through the matrix protein laminin and collagen IV for the secretion of lipolytic and glycosidic enzymes, along with proteases. The expression of spore coat homologs protein (CoTH) binding is done to the endothelial receptor glucose receptor protein 78, which provides fungal endocytosis into host cells.

MCR or mucorales can lead to a severe complication called angioinvasion, resulting in arterial thrombosis (42). In general, the mucorales tend to evade breaking down the extracellular matrix proteins laminin and collagen IV. The invasion by the host is related to the secretion of enzymes, i.e., lipolytic, glycosidic, and proteases, including aspartic proteases. The expression of spore coat homologs (CotH) proteins binds to the host endothelial glucose-regulator protein 78 (GRP78), which further acts as a fungal ligand for this unique receptor. The expression of CotH and GRP78 induces fungal endocytosis in the host cell resulting in invasion and cellular damage (42, 43), as summarized in **Figure 2**. An anti-GRP78 immune serum was found to protect mice with DKA from MCR, adding to the evidence of pathogenesis (7).

## 4 Risk factors for individuals prone to fungal disease

The susceptibility to COVID-19 varies among individuals and can be influenced by various factors, i.e., age, preexisting health conditions (e.g., diabetes, heart disease, and compromised immune system), genetics, and latent exposure to the virus (44, 45). Although anyone can contract COVID-19, the geriatric and individuals with underlying health issues are more susceptible to severe symptoms. There have been several reports of increased MCR cases among individuals recovering from COVID-19, particularly in India during the second wave of the pandemic. This has led to speculation that COVID-19 and its treatments might augment the risk of MCR (46). Various clinical representation also suggested the patients with DKA (47), immunocompromised, HIV/AIDS (2), organ transplantation (9), chemotherapy on corticosteroids (11), abraded tissues/necrosis, and wound-associated thrombosis are at risk for fungal infection. The MCR can occur or be seen within 2-3 weeks after recovering from COVID-19, especially in individuals with low counts of mono and poly-morphonuclear phagocytes (42). In a study, Ibrahim et al. reported that generating oxidative metabolites and defensins (cationic peptide) leads to rapid induction of NF-κB pathway-related genes via neutrophils in MCR (7). Recently, numerous cases of MCR in COVID-19 patients have been reported globally due to the increase in COVID-19 (approximately 3,50,000) cases daily. People with COVID-19 who have concomitant comorbidities, i.e., chronic obstructive pulmonary disease (COPD), diabetes, and immunosuppressive situations, including ventilation, corticosteroids, medication, and intensive care unit (ICU) patients, are more susceptible to life-threatening “opportunistic” infections (48). Several patients with COVID-19 frequently witness a surge in MCR instances, primarily attributable to the increased usage of steroids (e.g., dexamethasone), particularly by diabetic patients. The overuse of steroid medication can worsen the condition caused by black fungus, even in low-risk patients. Many factors are involved in the SARS-CoV-2 infection, including susceptibility, illness severity, and outcomes. The SARS-CoV-2 infection spreading and critical cases were less among the children, which showed age disparities. The clinical symptoms of SARS-CoV-2 are less common in children, while older adults (>65 years) are more likely to experience severe complications and morbidities (49). The presence of multiple infectious diseases and susceptibility is based on the host genetic background, while a few of the genetic variants are also related to SARS-CoV-2 (50, 51). It has been observed that the high rate of COVID-19 pathogenesis and mortality found in obesity and type 2 diabetes individuals contributed to a high consumption rate of saturated fats, sugars, and carbohydrates, collectively called the Western diet (WD) (52). There was a higher rate of fatality found in patients with comorbidities in COVID-19, as reported by the Chinese Center for Disease Control. In contrast, the fatality rate for the patients without comorbidities was around 0.9% (53). In another view, comorbidities-wise, the fatality rate was 7.3% in diabetes, 10.5% in cardiovascular diseases (CVDs), and 6.0% in hypertension (53). The risk of severe COVID-19 infection is nearly three times higher in obese individuals than in healthy population (54).

### 4.1 Mechanism of fungal infection associated with COVID-19

**Figure 3:**
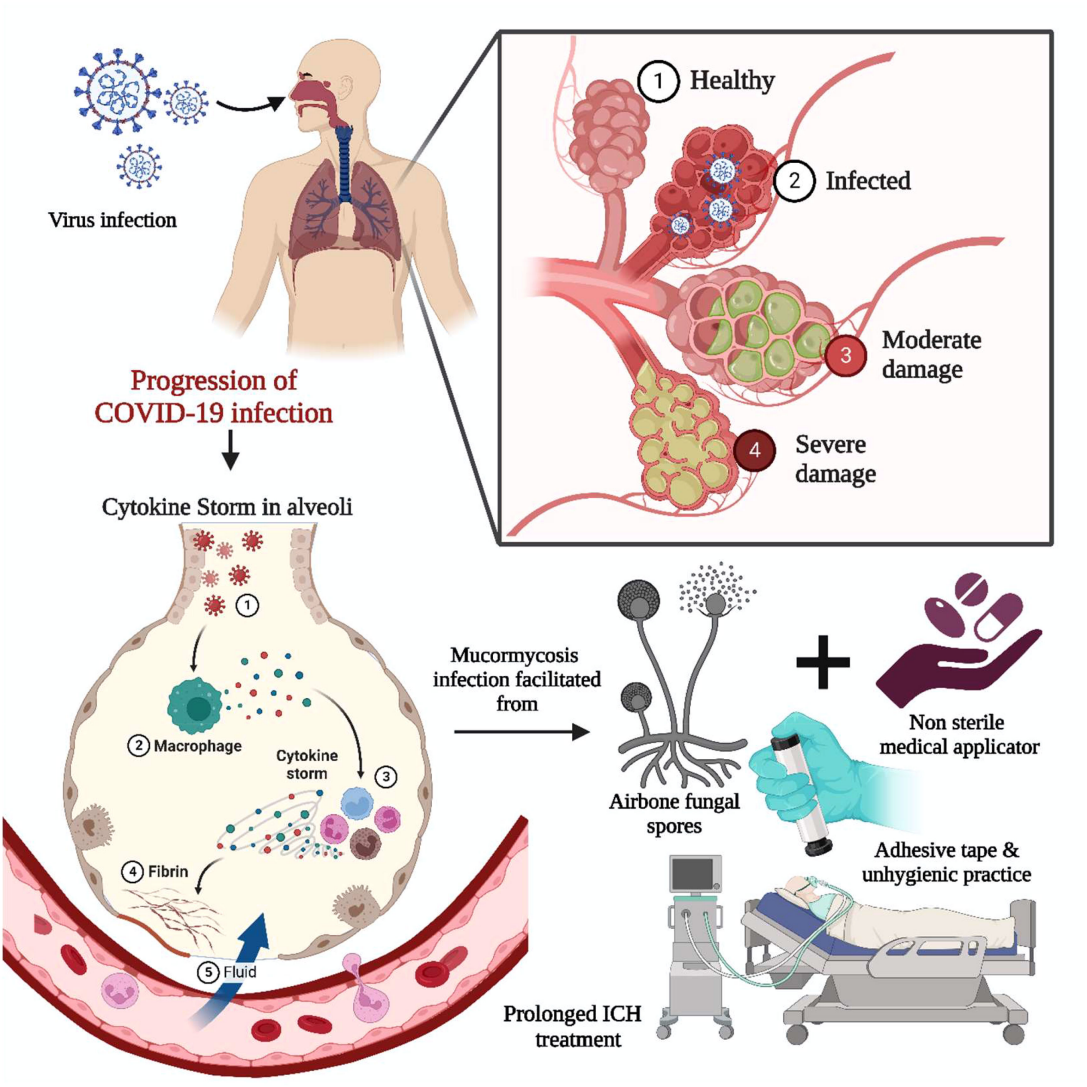
Impact of COVID-19 on MCR. The COVID-19 infection-induced acute respiratory syndrome involved the activation of interleukin and cytokine storms. The proinflammatory cytokine necrosis factor and immunocompromised state fueled the infection through airborne fungal spores. Prolonged bedridden conditions during the intensive care unit, exposure to unsanitary environmental conditions, non-sterile medicinal drug applicators, and adhesive tape are significant for primary cutaneous and other forms of mycosis.

The COVID-19 virus infects the respiratory system, causing severe inflammation of the lung tissues, which may lead to death. To alleviate lung inflammation in critically ill COVID-19 patients, doctors prescribe steroids and the antiviral drug Remdesivir©. However, the combination of steroids with Remdesivir© has been encountered to be the leading cause of mycosis in COVID-19 patients (55). Various corticosteroids such as dexamethasone, hydrocortisone, prednisone, and methylprednisolone have been observed to reduce lung inflammation symptoms in critically ill COVID-19 patients by suppressing immune activity when administered orally or intravenously (11, 25). When exposed to unsanitary environmental conditions, airborne fungal spores take advantage of the patient’s weakened immunity and potentially worsen the situation, causing fungal infections (29). The prolonged intensive care unit (ICU) patients combined with unhygienic practices, i.e., usage of contaminated, non-sterile medicinal drug applicators, surgical dressings, and adhesive tapes, have been reported as sources of primary cutaneous and other forms of mycosis, as summarized in **Figure 3**. In addition, mycosis is more familiar among post-COVID-19 patients with co-morbidities, i.e., renal failure and autoimmune disorders like rheumatoid arthritis, lupus, diabetes mellitus, and rheumatic fever (8). The individuals infected with COVID-19 often have lower levels of white blood cells (WBC) (56). However, in severe cases, there may be an increase in neutrophils, D-dimer, and urea levels, leading to severe acute respiratory syndrome. The activation of interleukins (IL-1β) by SARS-CoV-2 can trigger the production of other proinflammatory cytokines such as IL-6 and TNF-α. To reduce pulmonary inflammation, corticosteroids are typically administered to modulate cytokines, mainly interleukins (IL-1,4,6,8,12) and TNF-α (55).

An uncontrolled immune response, ‘cytokine storm’ or cytokine release syndrome (CRS), has been documented in COVID-19 patients with an induced level of IL-6 and depleted lymphocyte level due to the SARS-CoV-2 infection (57). The anti-interleukin-6 therapy has been proposed to attenuate the CRS, further investigated using a mouse model to check the host’s immunity against fungal pathogens (8). A higher lymphocyte count may be advantageous to the adaptive immune system and stimulate the formation of T cells specific for mucorales, which may aid in the control of invasive infection lymphopenia (58). Furthermore, COVID-19 virus causes long-term lymphopenia, which can increase the risk of transmitting invasive MCR (59).

### 4.2 Diabetes-related mycosis in elevated serum iron levels

Typically, MCR begins in the nasal and paranasal sinuses after inhaling black fungal spores. The presence of preexisting diseases lowers an individual’s resistance, making them potential hosts for invasive fungal infections, often seen in patients with type 2 diabetes mellitus (47). Mucorales fungi exhibit the capability to thrive in acidic environments via diminished chemotaxis and phagocytic ability. The compromised natural host cell response significantly contributes to the propagation of fungal infections within the system. In cases of MCR (Mucocutaneous Candidiasis and Reticuloendotheliosis) and diabetes, fungal infections primarily affect the rhino-orbital-cerebral region and the cutaneous and pulmonary areas. In diabetic patients, the invasive fungal infection shows significant aggressive signs, including facial pain and purulent nasal discharges (60, 61). Thus, the complications associated with diabetes related mycosis are challenging to address via conventional antiviral therapies (62). The patient with DKA has elevated serum iron levels in acidic conditions, influenced by transferrin, lactoferrin-binding proteins, and ferritin-binding proteins (10).

Ketoacidosis is a metabolic complication that can arise in individuals with diabetes, especially when diabetes is uncontrolled, or there is a lack of sufficient insulin levels. This condition is not directly associated with a transferrin binding defect or increased iron concentration in ketoacidosis patients. Several patients with ketoacidosis have elevated levels of ketone bodies, leading to the severe condition. This process is unrelated to capacitation induced by the keto-reductase enzyme. The enzyme keto reductase is not known to be generated by the pathogen *Rhizopus oryzae* or to cause any MCR-related issues. Ketoacidosis is primarily a result of the body’s inability to properly use glucose for energy, leading to the breakdown of fats and the production of ketone bodies. Pathogens like *Rhizopus oryzae* are not associated with developing ketoacidosis in patients (63, 64). In addition, the excessive glycosylation caused by hyperglycemia lowers the iron affinity of transferrin (65). The iron chelation property of transferrin is impaired from that of the low pH condition in the blood vessels, an acidic condition induced by accumulated ketone bodies like β-hydroxy butyrate (BHB). In addition, Iron and BHB favor the growth of the fungus. The *in vitro* endothelium injury and the augmented fungal invasion happened from the expression of the GRP78 along with CotH. The infection’s establishment is triggered by the host’s iron acquisition. The patients on hemodialysis and receiving the siderophore deferoxamine as a part of the iron overload treatment are predisposed to disseminated MCR (7). The drug deferoxamine prevents iron overload toxicity by removing excess iron from the host (65). These developments help to explore the importance of host iron acquisition for the pathogenesis of MCR and its contribution to fungal growth during host cell invasion—few of the research supported the significance of the serum iron levels in the MCR pathogenesis condition. The exploration of the liposomal amphotericin B and Deferasirox has demonstrated improved survival and reduced tissue fungal burden in the case of mice with DKA conditions. The Food and Drug Administration (FDA) recently approved Deferasirox as an iron chelator and can be incorporated into a liposomal delivery system against MCR (10).

## 5 Statistics on COVID-19 mycosis in diabetic and steroid patients

Recently, the Ministry of Health & Family Welfare, Govt. of India, has reported 28,000 active cases in India and declared a “black fungus epidemic” (26). Of those cases, about 8% of the active cases had a diabetes background, while 14.9 % suffered from DKA. Additionally, the survey revealed that corticosteroid treatment was administered to approximately 86% of patients. The use of corticosteroids in COVID-19 treatment has been extensively studied, with evidence supporting their use in some instances. However, the treatment protocols for COVID-19 are evolving rapidly, and it is essential to consult the most recent guidelines and research for up-to-date information, as recommendations can change over time (66). In most circumstances, patients with severe or critical COVID-19, especially those experiencing respiratory distress or requiring mechanical ventilation, were usually prescribed corticosteroids. However, it was generally advised against administering them in mild cases or during the early stages of the disease as they could impede the body’s natural ability to combat the virus. Dexamethasone, a type of corticosteroid, has demonstrated advantages in reducing mortality in hospitalized COVID-19 patients requiring oxygen or mechanical ventilation. The recommended dose was 6 mg once daily for up to 10 days (67). While dexamethasone was the most widely studied corticosteroid for COVID-19, other corticosteroids, i.e., prednisone and methylprednisolone, were also utilized in some instances. The choice of corticosteroids and dosage could vary based on the clinical situation and the latest guidelines. While beneficial in reducing inflammation, corticosteroids also have potential side effects, i.e., immunosuppression, hyperglycemia, and secondary infections. The use of corticosteroids is determined by evaluating individual cases and assessing potential risk factors and acquiescences (26, 68). In COVID-19 patients, the situation involves a scenario where 88.9% of cases experience sinus-related problems, while 56.7% encounter issues related to the rhino-orbital-cerebral region, where the mortality rate for these conditions was 30.7% (26). The opacification-related issues in the Sino maxillary and ethmoid sinuses were assessed in the many Computed tomography (CT) images of the face displaying mucosal thickening problems in COVID-19 patients (69). The pathogens that cause COVID-19-associated Mucormycosis (CAM) can withstand high temperatures and cause infection even in sterile conditions due to their thermotolerant characteristics. Numerous factors responsible for spreading the fungal disease in COVID-19 patients or survivors include endothelial damage, enhanced levels of ferritin, and multiplied levels of zinc and iron (69).

Diabetes mellitus (DM) is an independent risk factor for severe COVID-19 and MCR. The hyperglycemia at presentation (due to pre-existing DM or DKA) is the most critical risk factor observed in multiple cases (83.3%) of MCR with COVID-19, followed by cancer (26). The aberrant glycosylation of SARS-CoV-2, ACE2, and various immunoregulatory proteins, i.e., the Fc gamma receptor, may impact the severity of COVID-19 disease (70). A history of diabetes and hyperglycemia are independent risk factors for morbidity and mortality in SARS (71). Hyperglycemia, and not simply diabetes, may increase glycosylation of these proteins and lead to more severe COVID-19 disease. Various clinical reports indicated that prolonged uncontrolled hyperglycemia, rather than just a history of DM, may play a role in the pathogenesis of COVID-19 due to the binding of ACE2 by SARS-CoV-2 (70). The disease’s clinical symptoms involve nasal obstruction, swelling around the eyes or cheeks, and the appearance of black spots on the affected body area. The SARS-CoV-2 triggers the biochemical response cascade in the patient’s body, allowing fungal infection to thrive in an immunocompromised state (37, 72, 73). In addition, multiple studies have shown that COVID-19 infection can lead to invasive fungal disease by impairing the endothelial lining (73, 74). Recently, Bellanger and colleagues have proposed utilizing steroids as a treatment for SARS-CoV-2, potentially shaping its progression to resemble that of the suspected host. These developments also resulted in the growth of secondary fungal infections, which can lead to vision loss and hearing impairment, eventually resulting in mortality for COVID-19 patients (74). Thus, it is quintessential to investigate the cause along with the suggested treatment for the MCR to encounter the complications of the risk of COVID-related comorbidities (30).

Castro GS et al., have analyzed the MCR cases in patients previously diagnosed with COVID-19 from May 2020 to May 2021 in a single centre in Western Mexico (75). Most of the patients had diabetes (a total of 5 patients out of six) and received corticosteroid therapy; from the selected group of patients, only three suffered from severe COVID-19. After the proper analysis, the study estimated that COVID-19-associated mucormycosis was 300 times more frequent among the COVID individuals in the selected study region. The strict type of glycaemic control and the use of corticosteroids in non-severe COVID-19 cases helped prevent complicated cases of fungal infection (75). In another study, Bhanuprasad et al., explored the risk factors associated with the MCR COVID-19 pandemic into the second wave of infection (76). A total of 164 participants were assessed for risk factors, of which 132 cases belonged to MCR with COVID while 32 were non-COVID MCR. The later cases were treated as a control group for the study. The cohort study analyzed the impact of uncontrolled diabetes newly detected within a year. The alterations in the iron metabolism observed in COVID-MCR cases with the help of higher levels of serum ferritin and reduction into the C-reactive protein. Furthermore, researchers have extrapolated that interlinked risk factors like overuse of steroids and uncontrolled diabetes mellitus have a crucial impact on COVID-MCR cases, reasonable control of hyperglycemia, and appropriate use of steroids can be very beneficial for COVID-MCR prevention and further complications (76). In another work, the risk of hyperglycemia and steroid use on rhino-orbit-cerebral Mucormycosis (ROCM) was studied by Ponnaiah M et al., across India (77). They have compared many factors, including use of steroids, glycemic status, practices, and socio-demographics. Furthermore, the researchers have used a crude adjusted odds ratio involved with confidence intervals of around 95% through the logistic regression. A total of 267 cases have explored the associated risk of ROCM in post-COVID scenarios. The research group have concluded that hyperglycemic condition, irrespective of DM, along with the overuse of steroid, was crucial for the high risk of ROCM into COVID infection (77).

The steroid therapy is routinely used for the treatment of viral pneumonia. However, there is an event of patient’s predisposition to secondary fungal and bacterial infections involving mortality and morbidity. The risk of invasive fungal infections like aspergillosis and MCR could be higher in patients with immune dysfunction, receiving corticosteroid therapy or suffering from severe viral pneumonia (78). The increasing risk event of MCR was found notably in patients with immunosuppression, hyperglycemic, or acquiring steroid therapy (78). According to a case study conducted in Germany by Seidel D et al., a national survey was conducted to estimate the disease burden and clinical estimation of COVID-associated MCR cases (79). They assessed the clinical presentation of MCR related to COVID-19 in Germany with the help of German mycology networks and scientific societies. The research group also utilized the clinical information from the FunguScope®; a further study period was reported between March 2020 and June 2021, involving around 13 cases of MCR-COVID. Many factors, like an immunocompromised state owing to organ transplantation or malignancy, the diabetic condition, and the involvement of corticosteroid treatment, were involved in the selected study. The prevalence rate of COVID-19 in hospitalized patients was 0.67% and 0.58% in two selected study centres. The same is studied in intensive care units, and it was found to be 1.47%, 1.78% and 0.15% in three different study centres, thus provided evidence that the development and prevalence risk of MCR was higher in COVID-19 cases as compared to non-COVID cases (79).

## 6 Unique virulence traits of fungal pathogen

Typically, fungal pathogens use different virulence strategies to infect and colonize their hosts successfully, evading the host’s defences and causing damage. Different fungal pathogens may exhibit different combinations of these traits, and their virulence mechanisms can vary widely based on factors, i.e., host species, specific fungal species, and type of infection (80). The unique virulence traits of fungal pathogens iterate the importance of exploring the various virulence factors attributive to the infection (7). The general understanding of the mechanism of fungal attachment to its substrates, its physiological growth conditions in the host, and its mechanism of action is essential to devising drug formulations that can act against the fungal establishment in the host. Fungal pathogens first infect epithelial cells composed of the protein matrix, then gradually move towards endothelial cells, then reach the bloodstream and spread by hematogenous dissemination (81). The fungal sepsis can be induced due to fungi endocytosis into the host cell, while the endocytosis was accomplished with the help of spore coat homolog (CotH) protein. This later protein binds to the host endothelial receptor, namely GRP78 (82). Furthermore, Section 4.1 discusses the methodologies of virulence caused by high serum iron, ketone, and glucose levels.

**Figure 4:**
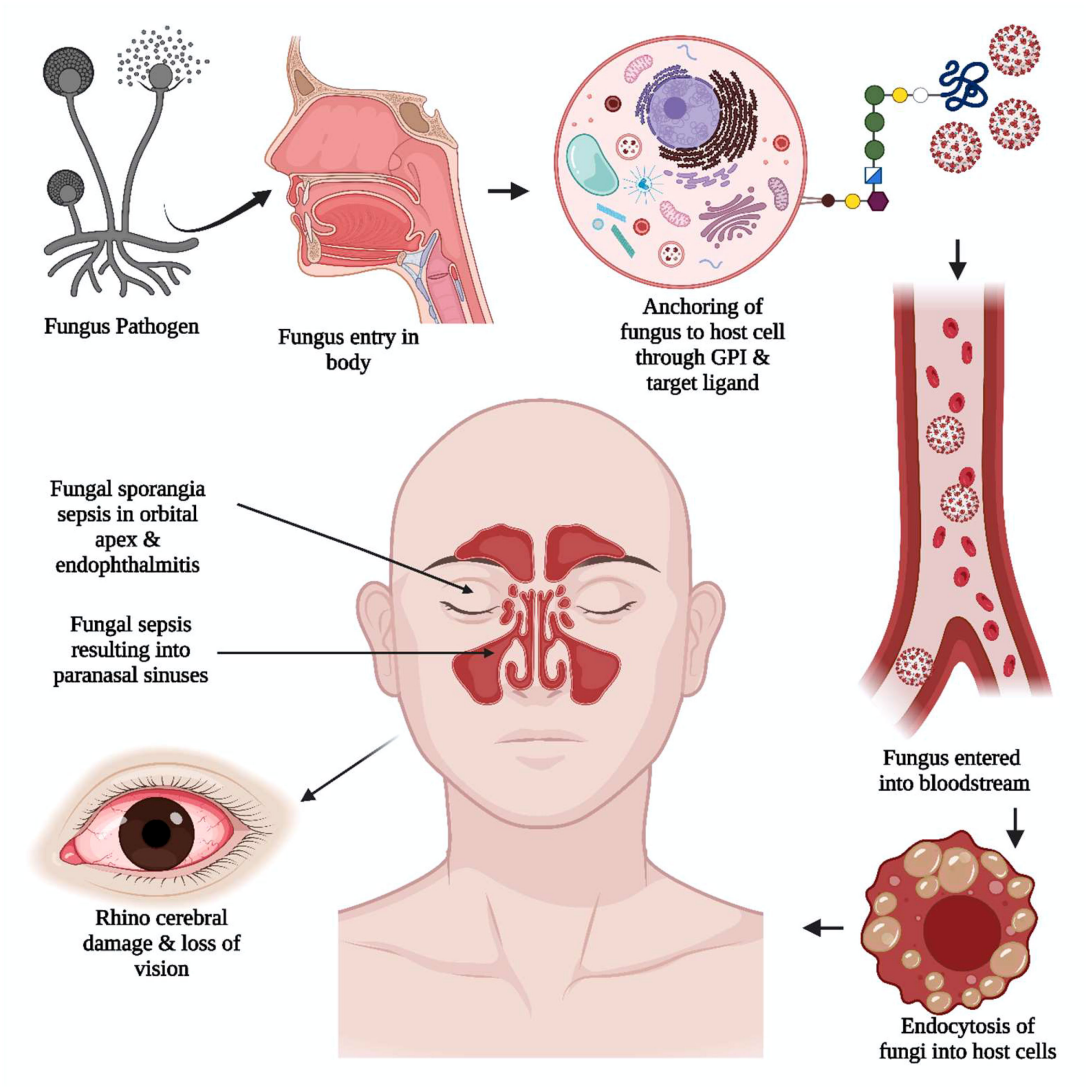
The unique viral pathogenic trait in black fungus. The anchoring of black fungus to the host cell is supported by glycosylphosphatidylinositol (GPI). The adherence of the pathogen to the host cell wall glycan and target ligand of the cell helped the pathogen get into the bloodstream. The fungal sporangia sepsis in the orbital apex, rhinocerebral damage, and endophthalmitis induce severe damage resulting in loss of vision.

In most instances, the disease is aggravated by the spread of fungal sporangia through the paranasal sinus to the orbital apex, causing endophthalmitis and loss of vision, ultimately resulting in rhino-cerebral damage (8), as illustrated in **Figure 4**. In many cases of mycosis before the COVID-19 pandemic, studies showed increased serum iron levels at the infection site. An increased incidence of MCR is observed in individuals with immune suppression and diabetic ketoacidosis (DKA) due to the elevated serum iron levels in DKA patients contributing to the fungi’s increased virulence (9, 10). Another significant factor that can increase virulence is fungal dimorphism, where the morphology of the fungus can change from a filament to a mold. The fungal dimorphism is influenced by factors such as temperature (83), the nutrient-rich micro-environment of the pathogen, and the host immune response (84). In most pathogenic fungi, i.e., *P. brasiliensis, B. dermatitidis, H.capsulatum, S. schenckii, P. marneffei*, and *C. immitis* of phylumAscomycota; *C. neoformans, C. gatti* of phylum Basidiomycota; filamentous morphology is found to exist at 20-25°C and yeast form occurs at 37°C (2).

In many cases, the pathogenicity in mould form could be attributed to the eukaryotic cell structure supporting virulence. The fungi *P. brasiliensis, B. dermatitidis, H. capsulatum, and C. immitis* exhibit dimorphism onset from mycelial form and produce 3 to 20 µm diameter conidia spherules. They affect the pulmonary tissues, leading to coccidioidomycosis. The mycelial cell wall contains ß- (1, 3)-glucan, whereas yeast cells have alpha- (1, 3)-glucan. Alpha- (1, 3)-glucan makes the cell wall rigid and morphologically dimorphic, conferring parasitic resistance to phagocytes (84).

## 7 Adhesive proteins in pathogenic fungi

Various studies on fungal substrate metabolites have shown a class of adhesive proteins in fungi that enable host substrate fixation and fungal dimorphism (85). The adhesins studied in some pathogenic fungi, namely *Candida albicans, Candida glabrata, Aspergillus fumigatus, Penicillium marneffei, Histoplasma capsulatum, Fonsecaea pedrosoi, Blastomyces dermatitidis, Coccidioides immitis, Paracpccodioides brasilensis* and *Sporothrix schenckii*. These have disclosed the presence of the glycosylphosphatidylinositol (GPI) dependent type of the cell wall proteins, further, promote anchorage to host cell wall glucans and target ligands of mammalian trans-glutaminases, epithelial cells, laminin (86), fibronectin, host-cell carbohydrates, fucose, endothelial cells, mannose, N-acetyl-glycosamine, and extracellular matrix proteins (10). Yeast flocculation (flo11p) and mucin reported in budding yeast of *Saccharomyces cerevisiae* are adhesive glycoproteins that play a crucial role in extracellular matrix interaction and cell differentiation (87). Morphogenesis in Cryptococcus, which is supported by the Cofilin 1 (Cfl1) adhesin, is regulated by the zinc finger transcription factor 2 (Znf2) (39). The fungal cell wall or membrane mainly comprises 1, 3-*β*- and 1,6-*β*-glucans, mannoproteins, ergosterol, and chitin. Some common glycan structures consist of four mannose residues like glucosamine in the fungal GPI anchors. The phosphor-ethanolamine bridge is required for the anchored protein to link to that of the third mannose residue of the GPI. One more mannose residue may be added to the common core (88). Various studies on GPI-P cell wall protein metabolites can create drug models that reduce fungal protein synthesis, leading to treatment goals (89).

## 8 Current therapy for MCR in post Covid-19 individuals

The conventional medications incorporate first-line antifungals amphotericin (a polyene antibiotic), 5-fluorocytosine (a nucleoside analogue) and azoles such as posaconazole, isavuconazole, fluconazole, itraconazole, ketoconazole, and miconazole (4, 90). In post-COVID-19 individuals with MCR, amphotericin is administered intravenously daily for up to eight weeks. Amphotericin B (Am-B) in a liposomal form is more effective than intravenous amphotericin B (91).

### 8.1 Antifungal therapy

There are no specific drugs available for the mucor species; thus, specific broad-spectrum antifungal agents are required to combat the infections. Amphotericin B is the most effective antifungal agent, but it has shown less efficacy against *Cunninghamella bertholletiae* and *Apophysomyces elegans*. Some mucorales strains have also developed specific drug resistance against conventional antifungal treatments (92). A successful method for treating MCR involves using Posaconazole (PCZ) and Isavuconazole (ICZ) (93). It is not recommended to use itraconazole and terbinafine in invasive MCR infections as they are only active against a few strains. PCZ can be implemented during the prophylaxis, but it is not a first-line therapy for black fungus infection (94). According to recent literature, polyene-caspofungin combination therapy has improved clinical outcomes compared to mere polyene therapy (95). Since the *R. oryzae* requires iron to develop and cause pathogenesis, inhibiting the iron uptake may be a good alternative for treating black fungus. In two recent preclinical studies, researchers studied the effectiveness of Posaconazole combination therapy in mice with MCR. The study reported that combining Posaconazole and Am-B improved the rate of neutropenic mice infected with *R. oryzae* (89).

### 8.2 Liposomal amphotericin B

The pathogenic fungal infections, i.e., zygomycosis, can be particularly dangerous as the dimorphic yeast forms penetrate the blood-brain barrier (BBB) and cause severe infections like rhino-orbital infections that can spread to the central nervous system. It is important to remark that the BBB only allows specific metabolites to pass through (22). Lipid-mediated diffusion of drugs across the BBB is possible only when the molecular weight should be < 400 Da and consists of less than eight hydrogen bonds (96) that can be achieved only with receptor-interceded or nano-liposomal carriers. Animal cells contain lipophilic membrane metabolites that enable both loaded and empty liposomes to cross the BBB effectively (96). Previous studies have shown that fluorescent or gold-labelled liposomes (both loaded and empty) can bind to the cell walls of pathogenic dimorphic yeasts and molds. Liposomal amphotericin B is the most effective medication against CAM, which could be attributed to its lipophilic nature and potential antifungal effects (97). Nanotechnology was deemed the first to have been used for antifungal therapy with the introduction of the lipid-based amphotericin B formulation in the 1990s. The efficacy of liposomal Am-B against MCR is due to its ability to directly interact with the glycolipids present in the GPI-P proteins of the fungal membrane that fix the fungi to host cells. Alternative formulations like Am-B colloidal dispersion and liposomal Am-B are presented in addition to the lipid formulation, which was safer and more effective than the conventional Am-B (98). The conventional Am-B is usually dispensed with the help of sodium deoxycholate into suspension form through the parenteral route. Thus, the therapeutic effectiveness of Amphotericin-B is limited due to renal toxicity and infusion-related complications (99). Nanoformulations can address these issues, but limited and expensive options, such as parenteral dosage forms, pose significant barriers for nano-derived products (95).

### 8.3 Salvage therapy

Salvage therapy is a successful alternative to control the infection if conventional antiviral treatments fail to provide bioavailability. The two most readily available primary alternative medications for treating the fungus infection are posaconazole and deferasirox. Many case studies and publications have revealed successful outcomes of the above medications without toxicity (95). While using deferasirox alone, the effect on the liver and kidney should be constantly monitored, and the dose should be optimized accordingly. As a salvage treatment, 20 mg/kg/Day for 2-4 weeks is advisable. In preclinical trials, Deferasirox toxicity is recorded after four weeks of treatment (100).

### 8.4 Adjunctive therapy

The reverse immunosuppression can be achieved if additional or adjacent treatment is introduced to control the infection. Granulocyte activity is enhanced against mucorales hyphae destruction with the help of granulocyte (macrophage) colony-stimulating factor [G(M)-CSF] or interferon (101). A new research approach proposes using nebulized antifungal drugs to improve MCR treatment. Nebulization increases the concentration of the drug at the site of infection, enhancing its efficacy and reducing systemic exposure and toxicity (12, 102). Recently, it has been unraveled that the Mucorales peptide CotH3, which binds to the endothelial cell receptor (GRP78), is connected to MCR endothelial invasion. GRP78-specific immune serum to block GRP78 cell receptors may shield diabetic mice against mycosis. This peptide-receptor interaction could represent a fresh move toward therapeutic research (103).

## 9 Diagnosis of MCR with nanotechnology

The field of nanotechnology offers precise possibilities for disease prevention, diagnosis, and treatment due to its unique biological and physicochemical characteristics (104). Gold nanoparticles have been researched commercially as probes for detecting specific nucleic acid sequences and are also documented as potent molecules for cancer and other diseases. Recent medical advancements may have improved the course of MCR, where Lipid-based formulations of Amphotericin B (LFAB) have been the cornerstone of the first treatment for MCR (105).

### 9.1 Fungal detection biosensors

Biosensors can detect fungal infections early by targeting nucleic acid sequences with high accuracy, sensitivity, and specificity. Biosensor technology is anticipated to significantly support fungal diagnostic research in the future, including several techniques not currently used in medical mycology. The biosensor technology can detect early-stage fungal infections and provides real-time monitoring and diagnosis of infectious diseases. The assessment of the patient response can be accomplished with the support of continuous data monitoring using biosensor technology. Diagnostic devices may transform chemical, physical, or biological facts into applicable analytical indicators (106). The sensor will only be helpful for gene analysis in the presence of a diverse set of gene sequences, indicating the organism’s presence. Biosensors can detect fungal infections early by targeting specific nucleic acid sequences, increasing accuracy, sensitivity, and specificity (107).

The required signal is induced from the biosensors with the aid of recognition and transducer components. Using a biological recognition system, the analyte concentration translates the vital biochemical information into the chemical or physical output signal. The transfer of the signal towards the electric output domain from that of the recognition system can be done with the help of a transducer. The transducer is also implemented in converting chemical concentration into electrical signals with the help of binding events. There are four commonly used systems for generating signals or transducing, including electrochemical, optical, piezoelectric, and thermometric. The antibodies act as high-affinity binding transducers, but their binding is irreversible and helps to generate a one-shot detector (108).

#### 9.1.1 Electrochemical biosensors

Different recognition elements, i.e., proteins and enzymes, are practical in the semi-qualitative analytical assessment conducted through electrochemical biosensors. This assessment also involves other recognition elements, i.e., antibodies and receptors. The electrochemical biosensors are essential for generating the output signals with the low detection unit and have significant applications in field analysis and clinical application. The change in the electrical current resulting from the electrochemical oxidation or the reduction from the electroactive species involved in the biochemical reaction can be measured using current base electrochemical biosensors. The biosensors use electrochemical and chemical signals to detect pathogens and other targets. Recently, Etefagh R et al., have implemented sensors based on nanoparticles and nanolayers of copper oxide to detect the *Aspergillus niger* (109). The copper oxide nanoparticles were synthesized via the sol-gel technique, while the spray pyrolysis was utilized for the nanolayer preparation. The prepared biosensors are used to detect the toxic gas produced by *Aspergillus niger* and to monitor decay (110–112).

#### 9.1.2 Optical biosensors

The interaction between the analyte and the surface of the substrate plays an essential position in the optical biosensors, which are utilized to detect this transformation in optical biosensors. The measurement of the change in the physical quantity of the analyte produces the adsorbed or emitted version of the light. Optical biosensors are used to study receptor affinity and to analyze the association and dissociation of chemical moieties involved in kinetic interactions. Optical biosensors are used for label-free detection of different chemical and biological substances for real-time measurement. Optical biosensors are also used to detect fungal biomarkers, employed for bacterial cell detection with the help of “Whispering gallery mode biosensors.” Antibodies, lectins, and immune receptors can be utilized as molecular receptors to modify the glass whispering gallery mode sensor to detect fungal biomarkers (113–115).

#### 9.1.3 Piezoelectric biosensors

The piezoelectric crystal is used as a sensor in the piezoelectric biosensors to record the affinity-driven interaction. The oscillations on the piezoelectric surface change when the analyte binds to it. Tombelli S et al., reported DNA-based piezoelectric biosensors for the detection of the amplicon of the gene from *Aspergillus flavus* and *Aspergillus parasiticus* (116). The expression of this gene is related to the production of aflatoxins, which are very potent liver carcinogens in several animal species and humans (116, 117).

### 9.2 Nucleic acid-based diagnostic tests to discern fungal infections

Various molecular-level diagnostics tests, i.e., conventional PCR, real-time PCR (RT-PCR), PCR using ITS and rDNA sections, nested PCR, direct DNA sequencing, and PCR-ELISA, have been explored to determine the level of fungal infections in various ailments (118). Since primers may be designed to delineate certain diseases and propose edges regarding diagnostic explicitness, sensitivity and repeatability of producing false-negative results remain the concern. Despite no FDA-approved methods, conventional PCR techniques are rapid and can improve sensitivity (119). A modified version of the “nested PCR” method has been developed to improve sensitivity and specificity (120). Furthermore, an alternative possibility is to accomplish a preliminary diagnosis using pan-fungal DNA amplification and further establish the presence of particular fungal species using target-specific primers (121).

### 9.3 Point-of-care tests

The POCT diagnostics offer great potential for initial disease treatment interventions. They can be classified into two types: (i) those that require minimal equipment and are easy to use, making them suitable for use in regions lacking modern laboratory equipment, and (ii) those that use modern techniques but are quick to complete and can be condensed into lab-on-a-chip (LOC) technology, which can be used in microfluidic devices (122). The WHO emphasizes the importance of portability, affordability, sensitivity, uniqueness, resilience, and consumer-friendliness regarding medical devices. Among these devices, lateral flow devices that use immune-chromatography techniques are currently the most promising in scientific mycology and are further utilized as POCTs in various clinical settings (123).

POCT stands for point-of-care testing, and examples of it include home pregnancy testing kits and blood glucose monitoring units. The immunochromatography used in medical oncology is also related to the POCT. Some commercial water flow devices are also related to POCT and used to detect cryptococcal capsular antigens. These properties are used for the detection of cryptococcal meningitis in significant populations in the world. The detection of the Aspergillus antigen germ tube-specific glycoprotein of the *Aspergillus* species is also done by commercial water flow devices. The presence of active invasive infection can be detected early from the antigens found in human serum or bronchoalveolar lavage (124–126). This test is not helpful in the detection of fungal spores as the fungal spores require proper germination and tissue invasion involved with synthesising glycoprotein targets. Many factors like lateral flow device, β-glucan, and galactomannan detection indicate that POCT can be used as adjunct tests for other biomarker tests required for detecting respiratory infections like pulmonary aspergillosis (127–130).

### 9.4 Detection of galactomannans

A renowned and historically well-marketed *Aspergillus* biomarker is Galactomannan (GM). GM is a 20-kilo Dalton polysaccharide in some fungal species, including *Aspergillus, Penicillium*, and others. In an immunoenzymatically sandwich microplate test for galactopyranosyl-containing substances, a rat-derived monoclonal antibody specific for Aspergillus is utilized despite cross-reactions with other fungal groups such as Fusarium. Patients with severe mucositis caused by aspergillosis have been reported to have galactomannan in their blood (131). To detect the disease early, the test may require higher sensitivity as high levels of GM are produced only in later stages of invasive aspergillosis, usually after angioinvasion. Using a higher cut-off index value, serum GM can predict outcomes and evaluate the effectiveness of antifungal medications (131).

## 10 Novel approaches to drug delivery

The antifungal medication has drawbacks, including the need to find the proper dosage, potential side effects from infusions, and a high risk of kidney damage, among other therapy-limiting effects. Recently, Am-B has been developed as a promising treatment option administered orally, topically, or via pulmonary routes (95). Due to their hydrodynamic size and straightforward administration, the lipid-based formulation of molecules like AmB or Nystatin might be enhanced to lessen the toxicity of conventional medications. Recent research has focused on using specific nanoparticles, namely silver nanoparticles (AgNPs), zirconium oxide nanoparticles (ZrO2NPs), and nanoemulsion NB-201, for their antifungal properties. These nanoparticles are being investigated because they can combat fungal infections (132). However, there is a concern associated with their use due to specific chemical components or “moieties” that can be potentially hazardous to human cells. The term “moieties” refers to the specific parts or components of these nanoparticles that could interact with cells. These interactions adversely affect human cells, making them hazardous (133). Therefore, while these nanoparticles are promising antifungal agents, researchers carefully evaluate their safety profile and potential risks to human health. The goal is to harness their antifungal effects while minimizing any harm they might cause to human cells or tissues (134). The MCR can be particularly challenging to treat due to the limited number of antifungal drugs that are effective against it. Posaconazole and isavuconazole are other antifungal agents that have demonstrated some activity against Mucorales and are sometimes used as alternative treatment options. However, their efficacy can vary, and they might not be as potent as desired against these infections (135–137). The first line of treatment for MCR is amphotericin B (also called amphotericin B deoxycholate, liposomal amphotericin B, amphotericin B lipid complex, and lipid-based amphotericin B) can have significant side effects, including kidney damage and electrolyte imbalances (97).

Moreover, resistance to antifungal drugs can develop, making treatment more challenging. MCR can rapidly progress and invade surrounding tissues, blood vessels, and organs. Surgical intervention is often necessary to remove infected tissue (138). Antifungal drugs may not effectively penetrate these infected tissues, limiting their ability to reach the site of infection. Achieving adequate concentrations of antifungal drugs at the site of infection can be difficult due to drug distribution and tissue penetration issues. This is particularly relevant in cases of MCR, where the fungus can infiltrate deep tissues (138). Lipid-based formulations of amphotericin B have a higher therapeutic index than the standard amphotericin B deoxycholate formulation. The therapeutic index measures a drug’s safety and effectiveness and is calculated as the ratio of the drug’s toxic dose to its therapeutic dose. A higher therapeutic index indicates a broader margin of safety and a more favorable balance between efficacy and toxicity. While effective in treating fungal infections, standard amphotericin B deoxycholate is associated with a higher risk of adverse effects, particularly nephrotoxicity (kidney damage) and infusion-related reactions (138). The narrow therapeutic index of the standard formulation means that the dosage needs to be carefully monitored to avoid toxicity. Lipid-based formulations of amphotericin B, i.e., liposomal amphotericin B and amphotericin B lipid complex in lipid bi-layer structure, lead to improve the drug’s therapeutic index (138).

### 10.1 New treatment methods

Various new approaches to treating host and fungal pathogens are continuously being developed. In addition to primary mono-therapeutic techniques, the discovery of new administration routes for aerosols to improve the treatment procedure is underway (105). Nanotechnology and antifungal microbial peptides are the future treatments for MCR. The encapsulated form is an oral formulation of AmB, which demonstrates high tolerance in studies on *Cryptococcus neoformans*; however, no information is available on its efficacy against MCR. Rezafungin, a more recent echinocandin, is yet to be tested in Mucorales. The genetic plasticity resulting from whole-genome duplication during evolution allows these fungal species to resist many antifungal agents, as demonstrated by *R. oryzae* genome sequencing (139). The antifungal drug VT-1161 is active against mucorales due to specific inhibitory action on the fungal CYP51 gene, as proven *in vitro.* This drug has been identified as the most effective curative for prophylactic and curative treatment in mouse models of *R. arrhizus*, *Lichtheimia*, and *Cunninghamella* species exhibiting neutropenia, increasing survival rates (140). A new triazole, SCH42427 (an active enantiomer of antifungal agent SCH39304), is effectual in murine models for its broad spectrum of activity (141). The sAPX001A is a post-translational modification agent that targets the Gwt1 protein in the glycosylphosphatidylinositol pathway, further demonstrated superior to AmB in phase 1 clinical trials regarding effectiveness (142). The drug can act as a prodrug for manogepix (MGX), which has a similar mechanism of action as fosmanogepix (APX001) and is very promising for treating pulmonary MCR (143).

### 10.2 GPI-P inhibitors

The Glycosylphosphatidylinositols (GPI-Ps) act as functional proteins in humans and are flexibly transported by the glycolipid called GPI. These GPI anchors in human blood enable the binding of the GPI-P protein to the plasma membrane of the target cells. GPI anchor proteins are encoded by the phosphatidylinositol glycan class A (PIG-A) gene (144). Interestingly, studies have found that pathogenic fungi adhesins are also composed of these GPI-Ps. This compatible virulence mechanism could easily fixate fungal proteins in the human host (145). This insight enables formulations that selectively inhibit fungal adhesins from ceasing fungal growth and spread through the bloodstream without affecting the host’s GPI-Ps. Protein synthesis inhibitors that are selective for fungal species are the best candidate drugs to treat pathogenic fungal infections without affecting mammalian cells. Sordarin is an antifungal compound isolated from the fermentation broth culture of the fungus *Sordaria araneosa* by scientists in Sandoz, Switzerland. Sordarins and their derivative compounds, such as GR135402, GM160575, GM191519, GM193663, and GM211676, are effective against *Candida* and *Cryptococcus* species in yeast form by targeting the elongation factors EF-1, EF-2, and EF-3 triggering total inhibition of fungal protein synthesis (146). Generally, sordarins block the function of yeast EF-2 and ribosomes, resulting in selective inhibition of the fungal protein elongation cycle in yeasts without affecting the host protein synthesis mechanism (146, 147). Sordarin acts on fungal proteins by inhibiting the translocation of ribosomes along with mRNA during the elongation of the nascent polypeptide chain (148). The bioactivity of sordarin against a dimorphic pathogenic fungus *Candida albicans* displays unusual yeast morphology change at 25°C unlike most fungal pathogens that exhibit fungal dimorphism at 37°C reveals its appropriate mechanism of action targeting the GPI-anchors and that GPI-Ps can be potentially expanded into a new antimycotic agent (2, 81).

### 10.3 Iron chelators

As mentioned earlier, elevated serum iron levels at infection-prone sites are a critical factor supporting the virulence of pathogens. Surplus iron at the site of infection feeds the iron requirement for pathogenic fungal cells, stimulating the growth of fungal pathogens. Carrier proteins such as transferrin, ferritin, and lactoferrin enable mammal iron absorption and balance. Reducing serum iron levels is one of the most common defense mechanisms of host cells against disease-causing pathogens such as bacteria and Mucorales because of their sustainability to survive only in elevated iron serum levels (149, 150). Various studies on the fungal pathogen *S. cerevisiae* have revealed that the mechanisms of iron uptake by reductive (88) and non-reductive mechanisms include siderophore-mediated uptake and heme acquisition (151). Furthermore, iron modulates the morphological differentiation of the opportunistic species by maintaining iron homeostasis to avoid toxicity (150, 152). The reductive iron uptake involves ferric reductases that reduce ferric iron to soluble ferrous iron. The process is mediated by FRE genes, FRE1, and FRE2, and reoxidation to the ferric form by a multicopper ferroxidase sequentially to be transported into the cell by a permease (153). Non-reductive iron uptake is achieved through ferric iron-specific chelators’ siderophores’ mobilizing iron. Free serum iron favors the expression of genes in fungal adhesins (153, 154). These findings indicate that iron chelators’ potential pharmacological action against fungal pathogens suppresses iron uptake metabolism (155). Several iron chelators, i.e., deferiprone and deferasirox, are encountered to inhibit fatal infectious fungal pathogens, especially *R. oryzae*, that impact MCR (10). Iron chelators should be avoided in dialysis patients due to the risk involved (156). A recent study found deferasirox effective in mice with DKA-associated MCR and wild *Drosophila melanogaster* flies infected with *R. oryzae*. Synergistic therapy with deferasirox combined with liposomal amphotericin B has improved the survival rate in a mouse model (55). Nevertheless, further clinical trials of synergistic treatments are required to treat patients with rhino-cerebral MCR.

#### 10.3.1 Role of turmeric as a natural iron chelator

**Figure 5:**
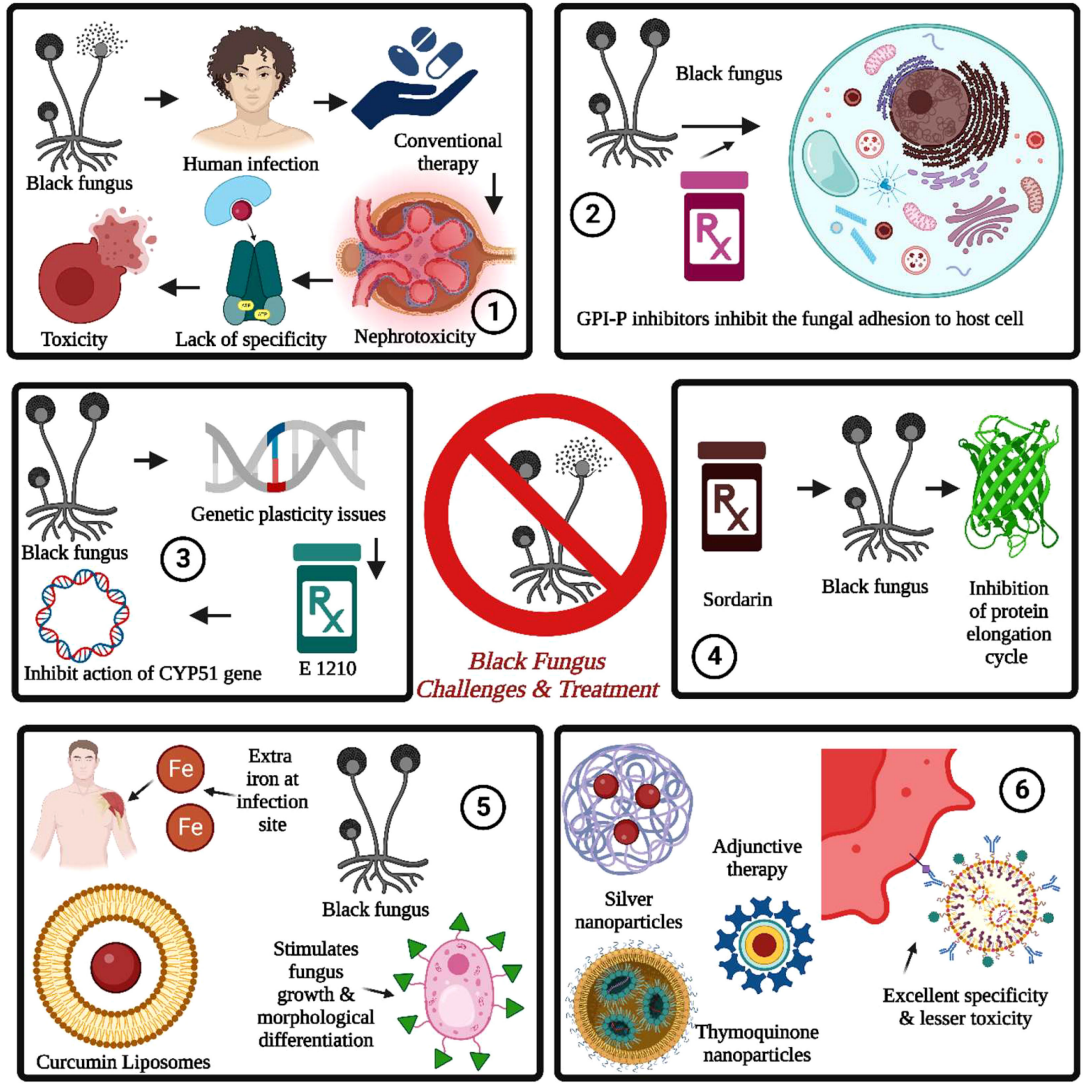
Black fungus challenges and treatment are summarized as follows: 1. The conventional therapy for black fungus suffered from nephrotoxicity, a lack of specificity, and toxicity issues. 2. The GPI inhibitors prevent fungal cell adhesion, followed by fungal growth and spreading into the bloodstream. 3. The fungal resistance can be induced through genetic plasticity issues induced by whole genome duplication. It can be addressed by E 1210. 4. The sordarins is used to inhibit the protein elongation cycle in black fungus by inhibiting the translocation of ribosomes and mRNA. 5. The iron chelators are used for inhibition of stimulation of fungal growth by the presence of iron at the infection site for the same. The nanotechnology-driven nanoparticles provide excellent specificity with lesser toxicity.

Turmeric is an iron chelator, and its combination with various phytochemicals synergistically helps to fight pathogens (157), binds to excess labile iron in the blood (158). Excess iron can be eliminated through chelation without disrupting cellular iron uptake. Animals experience extracellular iron chelation when iron is added to their diet (159). Nanoliposomes enriched with turmeric can function as iron chelators, replacing deferiprone and deferasirox. Curcumin, present in turmeric, decreases the H and L subunits of ferritin, the iron storage protein, in liver cells, which indicates a reduction in iron levels and the activation of iron-regulatory protein. Curcumin exhibits iron chelation in the liver, spleen, and bone marrow with no significant impact on plasma hemoglobin metabolism. Moreover, it has a harmless effect on the intestinal sections (160). Using turmeric liposomes shows promise in reducing serum iron levels by targeting specific organs and tissues such as the liver, spleen, lungs, and bone marrow. Curcumin is dispersed throughout an aqueous medium, which helps increase its efficiency. Nanocurcumin, on the other hand, has a higher intracellular absorption capacity, greater systemic bioavailability in plasma and tissues, and a significantly longer biological half-life than free curcumin. Consequently, it is considered a natural nano-iron chelator with great potential (161), as presented in **Figure 5**. The researchers have revealed the role of zinc as an anti-fungal element, particularly mucoromycetes. It has also been well demonstrated that it is difficult for fungi to survive when zinc is removed (162, 163). Due to the lack of such evidence at the time, unfortunately, zinc pills have been employed as immunity boosters during the COVID-19 pandemic. Overdosing zinc may harm the mucus lining of the GI tract and the wall of the gut epithelium. The lining of the GIT may consequently start to leak and develop prominent holes. Leaky gut walls may increase the levels of proteins, gluten, bacteria, and dietary antigens in the bloodstream, leading to intestinal inflammation, which sets off autoimmune disorders like IBD, celiac disease, and autoimmune hepatitis (164).

### 10.4 Thymoquinone-loaded nanoparticles

Thymoquinone (TQ) is an herbal candidate abundant in *Nigella sativa* that retains antioxidant, anti-inflammatory, antiviral (proven in hepatitis C, HIV, and other coronaviruses), antimicrobial, immunomodulatory, and anticoagulant potentials. Pulmonary damage results in inflammation due to the release of pro-inflammatory factors, i.e., interleukin-1β (IL-1β), interleukin-6 (IL-6), tumor necrosis factor-α (TNF-α), interferon-β (IFN-β), and prostaglandin E (PGE), which are encountered to be reduced by thymoquinone treatment in rats (165, 166). Pulmonary and intranasal drug delivery approaches mediated by nanocarriers have been proposed to increase bioavailability. Targeted delivery can be achieved through nano emulsions incorporated into inhalers and used as adjuvant therapies (3).

Furthermore, it can considerably reduce inflammation and enhance immunity in COVID-19 and mycosis-prone individuals to mitigate the effects of the virus. Antifungal activities of thymoquinone in aqueous extracts have been reported in *Candida albicans* (167); ethanolic extract activity is seen in Ascomycota (*Trichophyton rubrum*, and one each of *T. interdigitale, T. mentagrophytes, Epidermophyton floccosum*, and *Microsporum canis*) (168). The oil extract is effective against *Aspergillus parasiticus* and *Aspergillus flavus* (169), breaking ergosterol in the fungal membrane and leading to fungal death. TQ is a hydrophobic molecule, and its high encapsulation in nanoproduct can be obtained by employing the solvent evaporation method. Using biocompatible PLGA, the TQ-nan nanoparticle is fabricated to target the pulmonary airways with a 100–200 nm particle size. The PLGA hydrophilic surface enables better permeability and retention of nanocarriers in blood circulation. TQ-Nanopowder is more efficient than TQ-Nanosuspension in preventing premature drug release and enhancing thymoquinone’s permeability and retention (170). Liposomal thymoquinone (TQ) formulations are challenging due to TQ’s hydrophobicity. Dipalmitoyl phosphatidylcholine (DPPC) liposomes encapsulating non-polar TQ in the phospholipid bilayer can be exploited by nano drug design as it is one of the first studies describing TQ’s encapsulation in liposomes (171).

### 10.5 Silver nanoparticles

Several low-toxicity silver formulations were tested on human infections caused by mucorales species created from *in vitro* experiments (172, 173). Silver nanoparticles with the help of β cyclodextrin was used against *M. ramosissimus* (6, 174), could be further addressed as an antifungal strategy to halt the progression of MCR. Additionally, extensive research in a clinical setting is required to study the effects of silver nanoparticles on different species of mucorales. Nanoemulsion NB-201 uses benzalkonium chloride (BZK) surfactant to damage fungal cell membranes and kill the organisms as an antifungal treatment (105).

### 10.6 Other applications of nanotechnology in treatment of fungus infection

There have been increasing drug-resistant human pathogenic fungi cases over the past decades, and drug resistance to conventional antifungal therapy is one of the more significant challenges associated with the same. Numerous cases of high mortality have been linked to azole-resistant Aspergillus and Candida species, indicating insufficient protection for humans. The overuse of azole for treatment is one of the reasons for the development of these resistant species (175). The fungi have developed resistance mechanisms involving modifications of the drug targets, cellular pathways alterations, especially in sterol biosynthesis, reduction in the intercellular concentration of the target enzyme, and overexpression of the antifungal drug targets (176). The activation of the stress response signaling and overexpression of the efflux pump protein are also covered. There are some cases involved with acquired drug resistance in the fungi related to the stress response signaling, efflux pump protein overexpression, and target incompatibility due to biofilm formation changes into cellular permeability of the fungi into the host cells (177, 178).

Nanoparticles derived from nanotechnology show promise as advanced antifungal agents for forthcoming fungal diseases. Some of the properties of nanoparticles, including high specific surface area and physicochemical properties, are helping to kill the microbes. The nanoparticles have the potential to penetrate the biofilm and induce biofilm inhibition for the effective treatment of fungal biofilm infections (179–182). Metal-based nanoparticles are typically utilized to prevent fungal infections. For instance, gold nanoparticles can penetrate fungi and modify their genetic and metabolic processes, leading to the elimination of the fungi. The silver nanoparticles induce adhesion on the cell wall and membrane surfaces, penetrate cells to disrupt intracellular structures, induce cellular toxicity and oxidative stress, and modulate signaling transduction (183). In addition, copper and its alloys have excellent properties for effective antimicrobial action. The process involves releasing copper ions, electrically charged particles that prevent cell respiration, disrupt the microbe’s coat and destroy the microbial DNA and RNA. This process begins when microbes come into contact with the copper surface. Copper’s ability to prevent mutation in microbes helps to prevent drug resistance (184–187).

## 11 Therapeutics under clinical trial

Several therapeutics are currently being investigated for treating black fungus disease in clinical trials. There are several enduring trials around the world, including a pilot study on the effectiveness of high-dose liposomal Amphotericin B in treating initial Zygomycosis, Amphotericin B for pulmonary MCR, Deferasirox-AmBisome therapy for MCR, Isavuconazole for MCR and invasive Aspergillosis, Isavuconazonium Sulfate for treating invasive Aspergillosis or invasive MCR in children, and a post-marketing surveillance study of Cresemba© in Korea. Furthermore, **Table 2** summarizes various introspections of therapeutics under clinical trials. There is currently no specific vaccine available to stop this infection. It is advisable to avoid dusty environments such as construction or excavation sites. If it is necessary to be in such an environment due to work, wearing an N95 respirator is recommended (194, 195).

**Table 2:** Therapeutics under Clinical trials for the management of black fungus disease.

SI. No.	Title of the study	Drugs	Status	Clinical trial number	References
1.	Combined Inhalational with Intravenous Amphotericin B Versus Intravenous Amphotericin B Alone for Pulmonary mucormycosis.	Inhaled amp B deoxycholate+intravenous liposomal amp B and Intravenous liposomal amphotericin B alone	Recruiting	NCT04502381	(188)
2.	The Deferasirox-AmBisome Therapy for MCR (DEFEAT Mucor) Study	Deferasirox, Liposomal amphotericin B	Completed	NCT00419770	(189)
3.	Patterns of Real-World Isavuconazole Use - a Study of Patients with MCR or Invasive Aspergillosis	Isavuconazole	Not yet recruiting	NCT04550936	(190)
4.	A Study to Evaluate Isavuconazonium Sulfate for the Treatment of Invasive Aspergillosis (IA) or Invasive MCR (IM) in Pediatric Participants	Isavuconazonium sulfate	Recruiting	NCT03816176	(191)
5.	Pilot Study of High Dose Liposomal Amphotericin B Efficacy in Initial Zygomycosis Treatment	Liposomal Amphotericin B	Completed	NCT00467883	(192)
6.	Post Marketing Surveillance (PMS) Study of Cresemba in Korea.	Isauvuconazole group	Not yet recruiting	NCT04744454	(193)

## 12 Conclusion

The management and diagnosis of MCR infection is challenging, with indications that it may be spreading more rapidly than anticipated. In developed nations and those with uncontrolled diabetes, hematological malignancies are the most common underlying diseases. The clinical method of diagnosis needs to be more sensitive and specific. This paper describes the advancement of nanotechnology-based diagnosis of MCR (black fungus) and effective nano-based therapeutics for preventing this fungal infection. The importance of nanotechnology and the potential use of novel formulations such as liposomal curcumins, PLGA-coated TQ, Liposomal TQ, other iron chelators, and sordarins as promising GPI-P inhibitors have been discussed. To effectively manage the epidemic, public healthcare authorities should oversee the prudent use of steroids and antibiotics. Stopping the spread depends on raising awareness and getting a quick diagnosis. Deprived of prospective randomized controlled studies, adjuvant medicines are complicated to evaluate.

Moreover, there must be adequate resources for diagnosing mucorales. All COVID and post-covid patients should be in constant surveillance with the healthcare authorities to ensure the early assessment and detection of this lethal mucorales infection. Lastly, the government should establish black fungus detection and prevention departments in hospitals and must collaborate and encourage pharmaceutical companies to develop efficient antifungal medications.

## Author contributions

MF: Conceptualization, Formal Analysis, Writing – original draft. AS: Conceptualization, Formal Analysis, Writing – original draft. SA: Writing – review & editing. AG: Writing – review & editing. SG: Writing – review & editing. SM: Writing – original draft, Writing – review & editing, Formal Analysis. CK: Writing – review & editing. SN: Writing – original draft. AH: Writing – review & editing. MA: Writing – review & editing. MB: Writing – review & editing. MGA: Formal analysis, Data curation, Writing – review & editing. SR: Writing – review & editing. AR-M: Writing – review & editing. LS-M: Writing – review & editing. AM: Writing – review & editing. DB-A: Writing – review & editing. RS: Writing – review & editing.
